# Famine exposure in early life increases risk of cataracts in elderly stage

**DOI:** 10.3389/fnut.2024.1395205

**Published:** 2024-06-20

**Authors:** Jiayuan Feng, Hui Niu, Sijing Zhu, Wanwan Xiang, Xiaoxue Li, Yang Deng, Xu Xu, Wenfang Yang, Mei Chun Chung

**Affiliations:** ^1^Department of Obstetrics and Gynecology, Maternal and Child Health Center, The First Affiliated Hospital of Xi’an Jiaotong University, Xi’an, Shaanxi Province, China; ^2^School of Public Health, Xi’an Jiaotong University Health Science Center, Xi’an, Shaanxi Province, China; ^3^School of Public Health, Tianjin Medical University, Tianjin, China; ^4^Human Resources Department, The First Affiliated Hospital of Xi'an Jiaotong University, Xi’an, Shaanxi Province, China; ^5^Division of Nutrition Epidemiology and Data Science, Friedman School of Nutrition Science and Policy, Tufts University, Boston, MA, United States

**Keywords:** famine, childhood, adolescence, cataract, early-life exposure

## Abstract

**Background:**

Epidemiological studies have shown that early-life nutritional deficiencies are associated with an increased risk of diseases later in life. This study aimed to explore the correlation between famine exposure during the early stages of life and cataracts.

**Methods:**

We included 5,931 participants from the Chinese Longitudinal Healthy Longevity Survey (CLHLS) 2018 cross-sectional data in our study. Subjects were categorized into three groups by their age during the famine: adulthood group, school age famine exposure group, and teenage famine exposure group. Utilizing binary logistic regression models, we investigated the relationship between early-life famine exposure and cataracts.

**Results:**

Compared to the adulthood group, both the school age exposure group (OR = 2.49, 95%CI = 1.89–3.27) and teenage exposure group (OR = 1.45, 95%CI = 1.20–1.76) had a heightened risk of developing cataracts in elderly stage. And the sex differences in the impact of famine during early years on elderly cataract risk were observed, particularly indicating a higher risk among women who experienced childhood famine compared to men with similar exposure.

**Conclusion:**

Famine exposure during the early stages of life is associated with a heightened risk of developing cataracts in old age. To prevent cataracts in elderly individuals, particularly in females, measures should be taken to address nutritional deficiencies in these specific periods.

## 1 Introduction

Cataract is a pathological condition of lens opacity, which is prevalent among individuals aged over 60 years old (1, 2). Its prevalence varies globally across regions and age groups, with a global prevalence of approximately 17.20% (2). In China, the prevalence of cataracts among individuals over 50 years old is notably high, reaching 27.45% (3). By 2050, the estimated prevalence of cataracts is expected to reach 33.34% among individuals aged 45–89 years old, and the total of cataract cases will be more than double to 240.83 million in China (4). Cataracts are the primary reason of blindness and the second most prevalent cause for visual impairment worldwide (5). At present, the primary and effective treatment for cataracts is still cataract surgery, which imposes substantial socioeconomic burdens. For instance, the cost of such surgery can exceed twice the annual income of patients in rural China, significantly diminishing people’s quality of life (6). With China’s rapid aging population, the disease and economic burden of cataracts are expected to increase, posing significant challenges for both clinical and public health systems.

The famine occurred in 1959–1961 due to food shortages triggered by natural disasters, which is regarded as a “natural experiment.” Based on this period, it provided us with an opportunity to explore the enduring consequences of early nutritional deficiencies on individuals’ health (7).

The developmental origin hypothesis posits that nutritional deficiencies during early stages of life is linked to a heightened risk for diseases of later life (8, 9). Previous research has linked early-life famine exposure with various chronic conditions such as obesity, hypertension, diabetes, and metabolic syndromes (7, 10–13). The development of cataracts may be attributed to several factors, including nutritional deficiencies, infectious diseases, aging, chemical or drug-induced damage (6, 14). Based upon these observations, this study hypothesizes that individuals who experienced famine during their early years may have a higher risk of cataracts during their elderly age.

To our knowledge, no studies have established a correlation between famine and cataracts. Disclosing a potential correlation between famine experience during early age and cataracts is considerable in terms of understanding origins of the disease and guiding preventive strategies. This article is the first exploration to investigate the correlation between famine and cataracts, concerning school age and teenage famine exposures and utilizing the CLHLS 2018 cross-sectional data. We also conducted separate association analyses in both male and female groups to explore the possibility of sex-specific impacts of exposure to famine on cataracts. Furthermore, additional stratified analyses were performed to delve deeper into this relationship.

## 2 Methods

### 2.1 Study design and participants

Data used in the current study were drawn from the Chinese Longitudinal Healthy Longevity Survey (CLHLS). It is a public database that focuses on individuals aged 60 years and above. The aim is to gain a better understanding of the determinable factors of healthy aging among China’s aging population. It is a nationally representative survey using a multistage stratified cluster sampling approach across 23 of China’s 31 provinces. The survey initiated with a baseline examination in 1998, followed by subsequent surveys in 2000, 2002, 2005, 2008–2009, 2011–2012, 2014, and most recently in 2017–2018. Throughout these periods, trained workers systematically collected and assessed data using structured questionnaires. The specific details have been described before (15).

**Figure 1:**
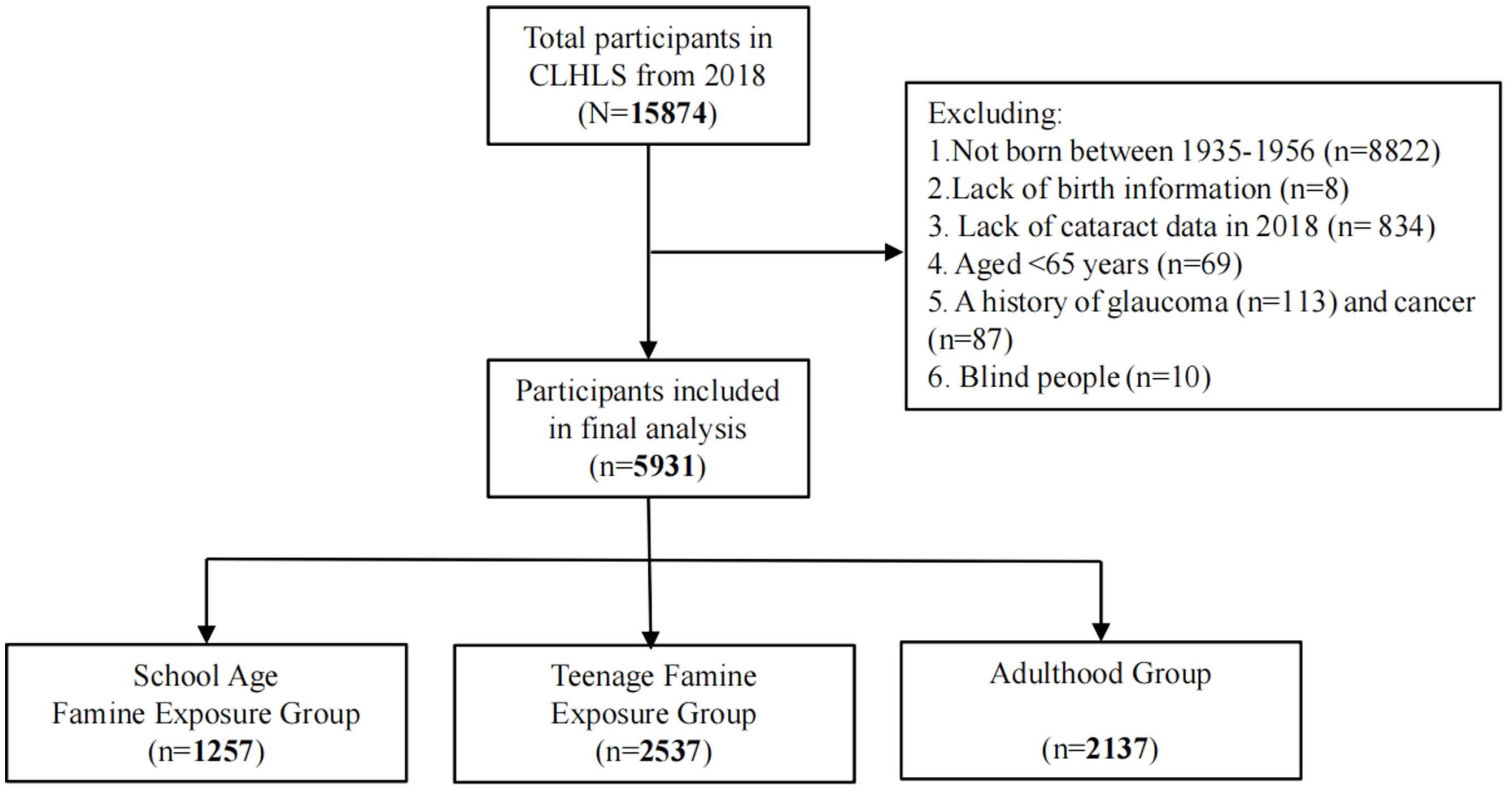
Flowchart of participants selected in the study.

The present study used the CLHLS 2018 cross-sectional data, and all individuals born from 1935 to 1956 were considered potential candidates. After screening based on specific inclusion and exclusion criteria, the study ultimately enrolled 5,931 participants in total (Figure 1).

### 2.2 Assessment of famine exposure

**Figure 2:**
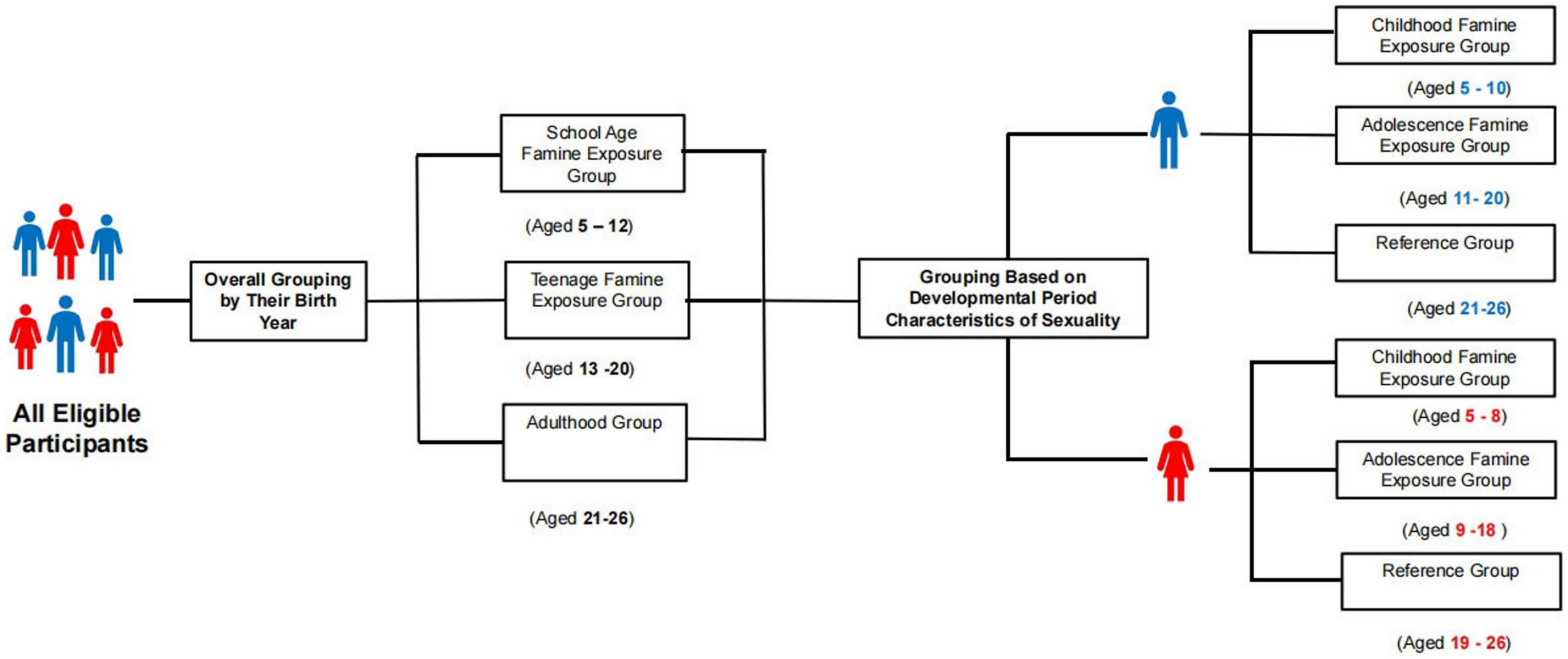
Flowchart of grouping after famine exposure assessment.

The famine exposure period was specified as ranging from 1959 to 1961. According to previous research and life cycle theory, school age and teenage periods are critical stages of growth and development, susceptible to nutritional deficiencies (16, 17). Adulthood follows the teenage period, marking completion of full physical development (18). We designate adults who experienced famine as the reference group and categorize subjects based on their age during the famine as follows: adulthood group (born between 1935 and 1940, aged 21–26 years old), school age famine exposure group (born between 1949 and 1956, exposed between 5 and 12 years old), and teenage famine exposure group (born between 1941 and 1948, exposed between 13 and 20 years old) (19, 20). Adolescence is a transitional period from childhood to adulthood with accelerated growth and development (21, 22). Considering these differences in the developmental period characteristics of sexuality, participants were further categorized as follows: (1) among male participants: unexposed group (born between 1935 and 1940, aged 21–26 years old), childhood famine exposure group (born between 1951 and 1956, exposed between 5 and 10 years old), and adolescence famine exposure group (born between 1941 and 1950, exposed between 11 and 20 years old); (2) among female participants: unexposed group (born between 1935 and 1942, aged 19–26 years old), childhood famine exposure group (born between 1953 and 1956, exposed between 5 and 8 years old), and adolescence famine exposure group (born between 1943 and 1952, exposed between 9 and 18 years old) (23, 24). The specific details showed in Figure 2.

### 2.3 Assessment of cataracts

Information about cataracts was collected by trained face-to-face interviewers. Participants were asked whether they had cataracts, either by responding personally or by a proxy through a relative. The response options were “1. Yes; 2. No; 3. Unknown.”

### 2.4 Covariates

Demographic, socioeconomic, and lifestyle variables were gathered as covariates. Education level was categorized as middle school or higher and primary or below. Marital status included both married and single (including divorced, widowed, or never married). Residence was classified as urban and rural. Ethnic group was divided as Han and Ethnic Minorities. China’s terrain descends from high in the west to low in the east, forming a three-tiered distribution (25). The first step includes regions with an average altitude above 4,000 m, the second step encompasses regions with average altitudes ranging from 1,000 to 2,000 m, and the third step constitutes regions with altitudes below 500 m (25). We divide the regional altitude into three steps based on each province’s geographical distribution, regional altitude was categorized into three classifications: (1) the first step (including Sichuan); (2) the second step (including Shanxi, Shaanxi, Heilongjiang, Beijing, Jiangxi, Hebei, Jilin, Fujian, Hubei, Guangxi, Hunan, Henan, and Chongqing); (3) the third step (including Tianjin, Shanghai, Zhejiang, Guangdong, Jiangsu, Shandong, Liaoning, Anhui, and Hainan) (26). Family income levels were defined as more than 50 k per year and less than 50 k per year. Smoking status encompassed both current smoking and non-smoking, while drinking status involved both current drinking and non-drinking. Exercise status was categorized as currently exercising regularly or not exercising currently. Finally, we used the excess mortality rates from 1959 to 1961 at the provincial level as an index to measure the famine severity (27). Areas with an excess mortality rate of 50% or more were considered severely hit by famine, while areas which with less than 50% were considered less severely hit by famine (28, 29).

### 2.5 Statistical analyses

To establish the baseline comparisons, categorical variables were presented by percentages and continuous variables were expressed by Mean and Standard Deviation. Utilizing the one-way ANOVA or Student’s *t*-test for continuous variables comparisons and employing the chi square test for categorical variables comparisons.

Employing logistic regression analysis, we evaluated the correlation between famine experience and the risk of cataracts, illustrated through crude and adjusted odds ratios (OR) and their respective 95% confidence intervals (CI). Initially, we implemented three models for an overall analysis. Subsequently, to explore the possibility of sex-specific impacts of famine experience on cataracts, further separate explorations were performed for both males and females. Finally, we performed stratified analyses based on various factors. However, due to the subsequent grouping and stratification, the sample size for each exposure group reduced, potentially limiting the findings for certain variables in the stratified analysis.

All the data were analyzed using IBM SPSS Statistics 25.0. Findings were determined with two-sided tests and considered statistically significant at *p* < 0.05.

## 3 Results

### 3.1 Characteristics of participants exposed to early-life famine

Table 1 presents the basic features of each participant based on cataract status. Out of the 5,931 participants, 707(11.9%) had cataracts. The study included 3,014 (50.8%) males and 2,917 (49.2%) females, and the mean age of all the participants was 74.4 ± 5.3 years. Significant differences were observed among gender, age, regional altitude, residence, smoking status, drinking status, exercise status, family income level, education level, famine severity, ethnic group as well as hypertension, diabetes, CHD, and arthritis between the groups with and without cataracts (all *p* < 0.05). Nevertheless, there were no differences within both groups in variables including marital status and the number of offspring (all *p* > 0.05).

**Table 1:** Baseline characteristics between subjects with and without cataract group.

Variables	Overall	Without cataracts	With cataracts	*p* values
*No. of participants, n (%)*	5,931(100.0)	5,224(88.1)	707(11.9)	**<0.001**
*Gender, n (%)*				**<0.001**
Male	3,014(50.8)	2,714(52.0)	300(42.4)	
Female	2,971(49.2)	2,510(48.0)	407(57.6)	
*Age (years), Mean (SD)*	74.4(5.3)	74.2(5.3)	76.0(4.9)	**<0.001**
*Regional altitude (km), n (%)*				**<0.001**
First step	464(7.8)	406(7.8)	58(8.2)	
Second step	2,609(44.0)	2,367(45.3)	242(34.2)	
Third step	2,858(48.2)	2,451(46.9)	407(57.6)	
*Residence, n (%)*				**<0.001**
Urban	3,253(54.8)	2,770(53.0)	483(68.3)	
Rural	2,678(45.2)	2,454(47.0)	224(31.7)	
*Smoking status, n (%)*				**0.019**
Current smoking	1,154(19.5)	1,043(20.0)	111(15.7)	
Non-smoking	4,777(80.5)	4,181(80.0)	596(84.3)	
*Drinking status, n (%)*				**0.017**
Current drinking	1,092(18.4)	987(18.9)	105(14.9)	
Non-drinking	4,839(81.6)	4,237(81.1)	602(85.1)	
*Exercise status, n (%)*				**<0.001**
Currently exercising regularly	2,403(40.5)	2,042(39.1)	361(51.1)	
Not exercising regularly	3,528(49.5)	3,182(60.9)	346(48.9)	
*Family income level, n (%)*				**<0.001**
More than 50 k per year	3,898(65.7)	1,709(32.7)	324(45.8)	
Less than 50 k per year	2,033(34.3)	3,515(67.3)	383(54.2)	
*Education level, n (%)*				**<0.001**
Middle school or higher	1,795(30.3)	1,526(29.3)	269(38.4)	
Primary or below	4,136(69.7)	3,698(70.7)	438(61.6)	
*Famine severity, n (%)*				**<0.001**
Serious	4,817(81.2)	4,334(83.0)	483(68.3)	
Less severe	1,114(18.8)	890(17.0)	224(31.7)	
*Ethnic group, n (%)*				**0.004**
Han	4,771(80.4)	4,186(80.1)	585(82.7)	
Ethnic minorities	1,160(19.6)	1,038(19.9)	122(17.3)	
*Marital status, n (%)*				0.296
Currently married	3,900(65.8)	3,453(66.1)	447(63.2)	
Currently single	2,031(34.2)	1,771(33.9)	260(36.8)	
*Of alive children, Mean (SD)*	3.4(4.0)	3.5(4.1)	3.3(3.6)	0.205
*Hypertension, n (%)*				**<0.001**
Yes	2,720(45.9)	2,290(43.8)	430(60.8)	
No	3,211(54.1)	2,934(56.2)	277(39.2)	
*Diabetes, n (%)*				**<0.001**
Yes	779(13.1)	623(11.9)	156(22.1)	
No	5,152(86.9)	4,601(88.1)	551(77.9)	
*CHD, n (%)*				**<0.001**
Yes	1,078(18.2)	858(16.4)	220(31.1)	
No	4,853(81.8)	4,366(83.6)	487(68.9)	
*Arthritis, n (%)*				**<0.001**
Yes	721(12.2)	525(10.0)	196(27.7)	
No	5,210(87.8)	4,699(90.0)	511(72.3)	

Data presented as number (%) for categorical variables and Mean (SD) for continuous variables. CHD, Coronary heart disease. *p* value for Student’s *t*-test (continuous variables) or Chi-squared tests (categorical variables). The bold values mean *p* < 0.05, showing significant statistical significance.

Table 2 displays these fundamental characteristics of the subjects categorized by their exposure to famine. 21.2% had experienced famine during school age, while 42.8% had experienced famine during the teenage years. Among different subgroups, significant differences were found for gender, age, smoking, and drinking status, family income level, education level, famine severity, ethnic group, marital status, number of offspring as well as diabetes, CHD, and arthritis (all *p* < 0.05). Nevertheless, there were no notable differences observed in regional altitude, residence, hypertension, and exercise status among the various subgroups (all *p* > 0.05).

**Table 2:** Baseline characteristics among different famine exposure groups.

Variables	Adulthood group	School age famine exposure group	Teenage famine exposure group	*p* values
*No. of participants, n (%)*	2,137(36.0)	1,257(21.2)	2,537(42.8)	**<0.001**
*Gender, n (%)*				**0.042**
Male	1,045(48.9)	635(50.5)	1,334(52.6)	
Female	1,092(51.1)	622(49.5)	1,203(47.4)	
Age (years), Mean (SD)	80.1(1.8)	66.9(1.4)	73.3(2.4)	**<0.001**
*Regional altitude (km), n (%)*				0.752
First step	173(8.1)	104(8.3)	187(7.4)	
Second step	930(43.5)	562(44.7)	1,117(44.0)	
Third step	1,034(48.4)	591(47.0)	1,233(48.6)	
*Residence, n (%)*				0.092
Urban	1,164(54.5)	723(57.5)	1,366(53.8)	
Rural	973(45.5)	534(42.5)	1,171(46.2)	
*Smoking status, n (%)*				**<0.001**
Current smoking	358(16.9)	251(20.2)	545(21.7)	
Non-smoking	1,779(83.1)	1,006(79.8)	1,992(78.3)	
*Drinking status, n (%)*				**<0.001**
Current drinking	326(15.5)	271(21.8)	495(18.7)	
Non-drinking	1,811(84.5)	986(78.2)	2,042(81.3)	
*Exercise status, n (%)*				0.129
Currently exercising regularly	831(39.4)	541(43.4)	1,031(41.2)	
Not exercising regularly	1,306(60.6)	716(56.6)	1,506(58.8)	
*Family income level, n (%)*				**0.020**
More than 50 k per year	750(35.1)	461(36.7)	822(32.4)	
Less than 50 k per year	1,387(64.9)	796(63.3)	1,715(67.6)	
*Education level, n (%)*				**<0.001**
Middle school or higher	510(24.0)	482(38.3)	803(31.7)	
Primary or below	1,627(76.0)	775(61.7)	1,734(68.3)	
*Famine severity, n (%)*				**0.020**
Serious	1,736(81.2)	989(78.7)	2,092(82.5)	
Less severe	401(18.8)	268(213)	445(17.5)	
*Ethnic group, n (%)*				**<0.001**
Han	1,602(75.0)	1,109(88.2)	2,060(81.2)	
Ethnic minorities	535(25.0)	148(11.8)	477(18.8)	
*Marital status, n (%)*				**<0.001**
Currently married	1,095(51.2)	1,009(80.3)	1,796(70.8)	
Currently single	1,042(48.8)	248(19.7)	741(29.2)	
*Of alive children, Mean (SD)*	4.0(4.1)	2.6(3.6)	3.4(4.1)	**<0.001**
*Hypertension, n (%)*				0.060
Yes	1,028(48.3)	542(43.3)	1,150(45.5)	
No	1,109(51.7)	715(56.7)	1,387(54.5)	
*Diabetes, n (%)*				**0.003**
Yes	265(12.5)	194(15.5)	320(12.7)	
No	1,872(87.5)	1,063(84.5)	2,217(87.3)	
*CHD, n (%)*				**<0.001**
Yes	437(20.6)	179(14.3)	462(18.3)	
No	1,700(79.4)	1,078(85.7)	2,075(81.7)	
*Arthritis, n (%)*				**0.049**
Yes	281(13.3)	153(12.2)	287(11.4)	
No	1,856(86.7)	1,104(87.8)	2,250(88.6)	
*Cataract, n (%)*				**<0.001**
Yes	331(46.8)	283(40.0)	93(13.2)	
No	1,806(53.2)	974(60.0)	2,444(86.8)	

Data presented as number (%) for categorical variables and Mean (SD) for continuous variables. CHD, Coronary heart disease. *p* value for ANOVA (continuous variables) or Chi-squared tests (categorical variables). The bold values mean *p* < 0.05, showing significant statistical significance.

### 3.2 Association of famine exposure in early life stage with cataracts in elderly stage

Table 3 demonstrates the correlation between experience of famine and cataracts using binary logistic regression. In Model 1, without adjusting any variables, the ORs of cataracts were 2.29 (95% CI, 1.80–2.92, *p* < 0.001) for the school age famine exposure group and 1.46 (95% CI, 1.23–1.73, *p* < 0.001) for the teenage famine exposure group compared to the adulthood group. Model 2 further adjusted for gender, family income level, education level, marital status, along with number of offspring, ethnic group, famine severity, regional altitude, as well as residence. The ORs of cataracts were 2.60 (95%CI, 2.00, 3.37, *p* < 0.001) for school age famine exposure group and 1.49 (95%CI, 1.24, 1.79, *p* < 0.001) for teenage famine exposure group, in comparison to adulthood group. In Model 3, the fully adjusted ORs of cataracts were 2.49 (95% CI, 1.89–3.27, *p* < 0.001) within the persons who were in the school age exposure group and 1.45 (95% CI, 1.20–1.76, *p* < 0.001) for the teenage exposure group, in comparison to adulthood exposure group.

**Table 3:** Binary logistic regression analysis the relationship between famine exposure and cataract among different groups.

Group	Model 1	Model 2	Model 3
	OR (95% CI)	OR (95% CI)	OR (95% CI)
	*p*	*p*	*p*
Adulthood group	Ref	Ref	Ref
School age famine exposure group	2.29(1.80,2.92)	2.60(2.00,3.37)	2.49(1.89,3.27)
	**<0.001**	**<0.001**	**<0.001**
Teenage famine exposure group	1.46(1.23,1.73)	1.49(1.24,1.79)	1.45(1.20,1.76)
	**<0.001**	**<0.001**	**<0.001**

OR, Odds ratio; CI, Confidence interval.

Model 1: un-adjusted. Model 2: adjusted for gender, family income level, education level, marital status, number of offspring, ethnic group, famine severity, regional altitude, and residence. Model 3: adjusted for variables in model 2 plus hypertension, diabetes, coronary heart disease, Arthritis, drinking status, smoking status, and exercise status. The bold values mean *p* < 0.05, showing significant statistical significance.

Table 4 illustrates the correlation between famine exposure and cataracts using binary logistic regression by gender. For the childhood famine exposure groups in Model 1, the ORs of cataracts were notably higher in the females exposed during the period of childhood (OR = 4.71, 95%CI: 1.47–15.10, *p* = 0.009) compared to the males exposed during the period of childhood (OR = 3.54, 95%CI: 2.05–6.11, *p* < 0.001). And Similar findings could be obviously observed in Model 2 and Model 3, consistently showing higher ORs of cataracts in the female childhood exposure group compared to their male counterparts in the same exposure group. For the adolescence famine exposure groups in Model 1, it was shown that the ORs of cataracts were 1.68 (95% CI, 1.35–2.08, *p* < 0.001) in females, and in males it was 1.74 (95% CI, 1.36–2.23, *p* < 0.001), both as compared to the unexposed group. Similar results were observed in both Model 2 and Model 3.

**Table 4:** Binary logistic regression analysis the relationship between famine exposure and cataract among different groups by gender.

Group	Model 1	Model 2	Model 3
	OR (95% CI)	OR (95% CI)	OR (95% CI)
	*p*	*p*	*p*
Men			
Unexposed group	Ref	Ref	Ref
Childhood famine exposure group	3.54(2.05,6.11)	4.72(2.65,8.40)	4.01(2.21,7.26)
	**<0.001**	**<0.001**	**<0.001**
Adolescence famine exposure group	1.74(1.36,2.23)	1.93(1.47,2.53)	1.77(1.33,2.35)
	**<0.001**	**<0.001**	**<0.001**
Women			
Unexposed group	Ref	Ref	Ref
Childhood famine exposure group	4.71(1.47,15.10)	5.72(1.75,18.72)	6.21(1.85,20.81)
	**0.009**	**0.004**	**0.003**
Adolescence famine exposure group	1.68(1.35,2.08)	1.71(1.35,2.17)	1.84(1.43,2.38)
	**<0.001**	**<0.001**	**<0.001**

OR, odds ratio; CI, confidence interval.

Model 1: un-adjusted. Model 2: adjusted for gender, education level, family income level, marital status, number of offspring, ethnic group, famine severity, regional altitude, residence. Model 3: adjusted for variables in model 2 plus hypertension, diabetes, coronary heart disease, Arthritis, drinking status, smoking status, and exercise status. The bold values mean *p* < 0.05, showing significant statistical significance.

### 3.3 Association of famine exposure with cataracts by subgroup

**Figure 3:**
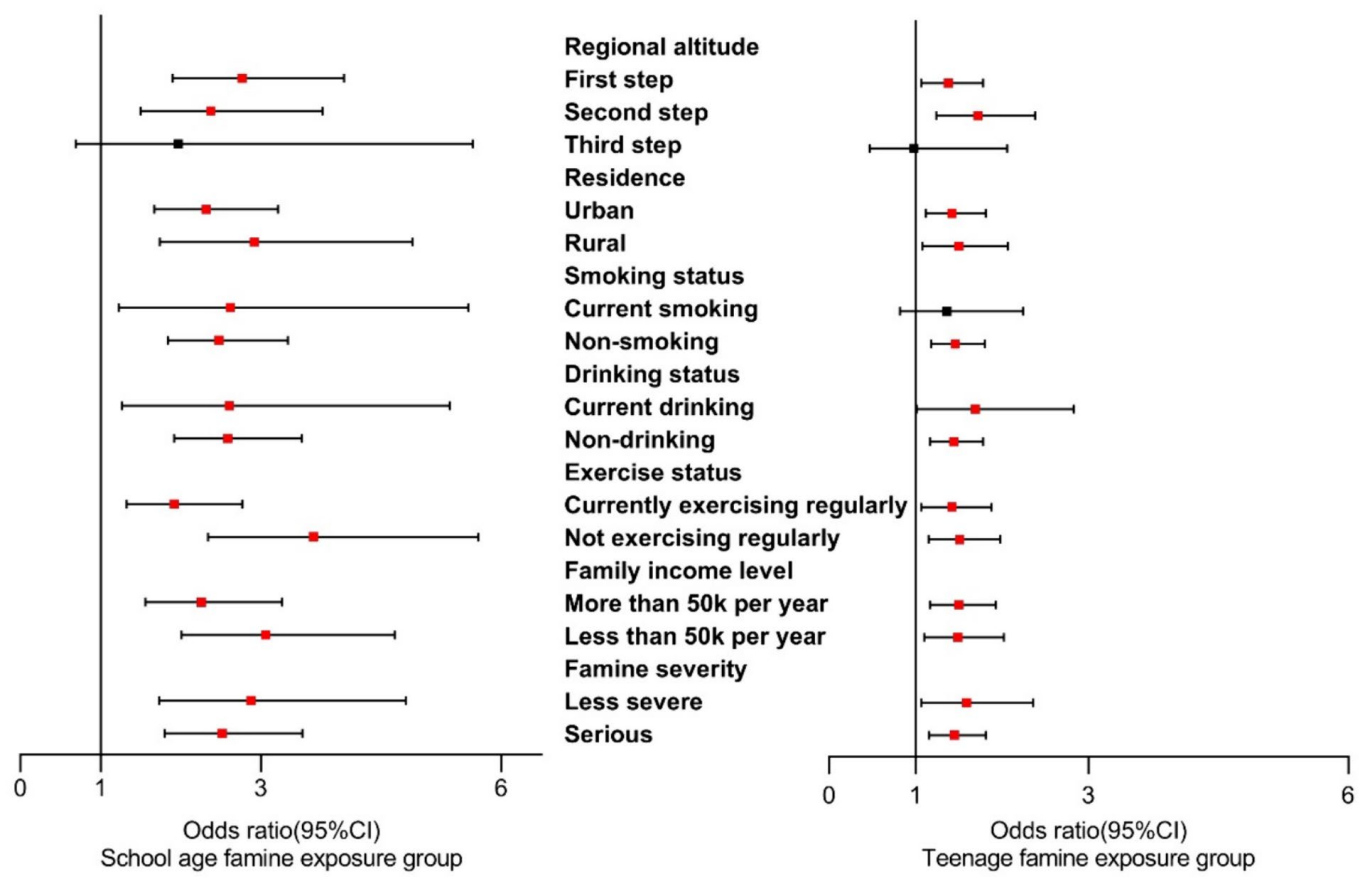
Stratified analysis on associations between famine exposure and cataracts in overall grouping.

Figure 3 displays the correlation between famine exposure and cataracts through stratified analysis. Our findings highlighted a significant relationship between school age exposure and cataracts among individuals in the first and second steps of regional altitude. Notably, school age exposure demonstrated a consistent relationship with cataracts across various subgroups, regardless of residence, smoking status, drinking status, exercise status, family income level, and famine severity. Teenage famine exposure exhibited a significant association with cataracts among individuals in the first and second steps of regional altitude, as well as non-smokers. Similarly, teenage exposure indicated a significant association concerning residence, drinking status, exercise status, family income level, and famine severity. Similar associations were found between famine exposure and cataracts among male participants, as depicted in Supplementary Figure 1. Additionally, Supplementary Figure 2 illustrates the specific relationship between famine exposure and cataracts among female participants.

## 4 Discussion

According to our knowledge, it is the first exploration about the correlation of famine exposure during the early stages of life with cataracts, specifically focusing on school age and teenage period. Our findings indicate a significant correlation between exposure to early-life famine and cataracts as adults, markedly stronger when exposed during school age compared to teenage period. Notably, sex differences were observed, with females exhibiting a greater correlation between childhood exposed famine and cataracts compared to males. Overall, individuals who have a history of experiencing famine are more susceptible to have cataracts when residing in higher altitudes, rural areas, among smokers, alcohol consumers, individuals with infrequent exercise, and those with lower income levels.

### 4.1 Correlation between famine exposure and cataracts

Prior to this, there were no specific studies on famine exposure and cataracts. Only one study suggests a potential connection between famine exposure and eye diseases (30). The finding shows an association between early-life famine exposure and multimorbidity in adults aged 65–71 years, with a significantly increased OR for multimorbidity (OR = 1.39, 95% CI: 1.04–1.87), defined as the presence of two or more chronic diseases out of 14 chronic conditions, including hypertension, eye diseases (cataracts, retinitis pigmentosa, glaucoma, macular degeneration, and diabetic retinopathy), heart diseases, and others (30).

Famine deprives school-age children and teenagers of sufficient nutrients during their periods of rapid growth and development, whereas studies show that high protein intake decreases cataract risk and increased polyunsaturated fat intake lowers the risk of cortical cataracts (31–33). Low protein intake may also indicate a deficiency in specific amino acids required for maintaining lens health, such as tryptophan, which can subsequently lead to lens damage (34, 35).

Most current research on nutrition and cataracts focuses on antioxidant vitamins (36) Firstly, multiple prospective studies indicate that increased vitamin C intake had a long-term protective impact against age-related cataract development (37–39). Participants in the highest quintile of total vitamin C intake (including dietary supplements) had a lower risk of nuclear cataract (OR = 0.55, 95%CI: 0.36–0.86) (37). Oxygen free radicals, formed during normal bodily metabolism, can induce oxidative stress linked to cataract development (40–43). These radicals, including superoxide, hydrogen peroxide, and hydroxyl radicals, can damage lens components (crystalline proteins, lens fibers, and lipids), potentially accelerating the development of nuclear cataracts (37, 40, 44). Vitamin C, a potent antioxidant that scavenges ROS, exists in high concentrations in the human lens, aqueous humor, and vitreous body (45, 46). This may explain why supplementing with vitamin C can delay the development of cataracts.

Secondly, a hypothesis proposes that antioxidant nutrients may protect against age-related lens damage (47). While most epidemiological studies have not provided evidence for vitamin E’s active role in preventing age-related cataracts, one study suggests reduced cataract risk with vitamin E intake from food and supplements, with a multivariate relative risk of 0.86 (95% CI: 0.74–1.00) (48–52). Vitamin E, with its antioxidant capabilities, interacts with selenium and glutathione peroxidase to prevent the formation of oxidation products of polyunsaturated fatty acids and oxidative damage, thus reducing cataract risk (53, 54).

Moreover, carotenoids may reduce the risk of cataracts, particularly lutein and zeaxanthin, which have been shown that these nutrients might have beneficial impacts in decreasing the risk of cataract formation (39, 48). Carotenoids, involved in forming cellular membrane components, maintaining membrane integrity, and facilitating regulated substance transport, can impact the development of posterior subcapsular cataracts if the integrity of the lens’s outer layer membrane is compromised (39). Additionally, other nutrients such as riboflavin, tryptophan, and calcium are being explored for their potential roles (34, 55–57).

Therefore, malnutrition and inadequate intake of essential nutrients during famine might contribute to cataract development (31, 32, 37–39, 48, 58, 59). However, further research is crucial to comprehensively grasp the relationship between nutrient deficiencies and cataract development.

### 4.2 Correlation differences among exposure group

Our results reveal that experiencing famine in the stage of childhood and adolescence heightens the risk of cataracts during the adult age. This aligns with previous research suggesting that individuals in childhood and adolescence may be more vulnerable to famine-related stress than in infancy. Consequently, famine experiences in the stages of childhood and adolescence potentially elevates the likelihood of cataract development during the adult stage (16). One potential explanation could be that individuals enduring famine in extremely early stages of life have opportunities to overturn the effects of nutritional deprivation and attain subsequent catch-up growth. However, childhood and adolescence are critical stages in the process of growth and development, potentially leading to irreversible impacts on long-term health (60).

Therefore, focusing on childhood and adolescence could be crucial in implementing interventions to improve elderly life quality and prevent chronic diseases.

### 4.3 Sex-based correlation differences

Our research indicates that childhood exposure impacts females more significantly than males. These sex differences might originate from several factors. Firstly, the son preference in traditional Chinese culture might contribute to better health outcomes for boys, as parents prioritize shielding boys from harsh environments and ensuring their access to sufficient food and nutrition (61, 62). Secondly, female health seems more susceptible to the influence of childhood conditions compared to male health, possibly due to different adaptations to early-life events between genders or biological differences in assumed social roles (63–65). Moreover, existing studies generally indicate higher cataract prevalence in females than males (2, 66–70). The only global study found female prevalence at 33.67% (95% CI: 25.90–41.44) and male at 32.57% (95% CI: 26.29–38.85) (2). A US study similarly found higher age-adjusted cataract prevalence in females than males (OR = 1.37; 95% CI, 1.26–1.50) (70). Some studies suggest that female gender is a cataract risk factor due to this gender disparity (2, 66–70).

### 4.4 Differential results from stratified analysis

UV radiation, excessive smoking and alcohol intake are the significant risk factors for age-related cataracts (71, 72). As altitude increases, the atmosphere’s capacity to filter UV rays diminishes, intensifying UV radiation that could potentially harm the lens through thermal and photochemical effects (73). Research has established a direct link between smoking quantity and cataract formation (74). Smoking affects cataract development by inducing oxidative stress on the lens through exposure to tobacco smoke, potential generation for reactive advanced glycation end products, and the direct toxic effects of heavy metals like cadmium, copper, and lead present in tobacco smoke (75). A meta-analysis highlighted that heavy drinking notably amplifies the probability of age-related cataracts (76). Alcohol consumption increases the hazard of developing nuclear, cortical as well as posterior subcapsular cataract due to the lens’s high vulnerability to oxidative stress and alcohol’s toxic effects (77). Therefore, maintaining a healthy lifestyle serves as a crucial factor in reducing the risk of developing cataracts.

### 4.5 Limitations

This population-based study displayed that famine exposure significantly heightened the risk of cataracts among the aged population. Nonetheless, it does have several limitations. Firstly, it was not feasible to use a cross-sectional design to establish a causal correlation between famine exposure and cataracts. Secondly, the relying on self-reported cataract data may introduced potential recall bias. Thirdly, the CLHLS 2018 cross-sectional data lacked sufficient participants born after 1956, hindering the analysis of fetal and infant exposure’s impacts on cataracts. Additionally, focusing on famine survivors in this study may lead to an overestimation or underestimation of famine effects. Finally, the lack of research on famine exposure and cataracts necessitates further investigation in the future.

## 5 Conclusion

Exposure to famine during early stages of life is correlated with a significant increased risk of cataracts during the elderly age. The study indicates the status of nutritional deficiencies during school age and teenage period has enduring impacts on people’s health outcomes during the elderly stage. To prevent cataracts in elderly individuals, particularly in females, measures should be taken to address nutritional deficiencies in these specific periods. It also highlights the significance of concentrating on nutritional intake throughout school age and teenage period, emphasizing the importance of promoting nutrition awareness within society.

## Data availability statement

Publicly available datasets were analyzed in this study. This data can be found at: https://opendata.pku.edu.cn/.

## Author contributions

JF: Conceptualization, Data curation, Formal analysis, Methodology, Writing – original draft, Writing – review & editing. HN: Conceptualization, Data curation, Formal analysis, Methodology, Writing – original draft, Writing – review & editing. SZ: Methodology, Writing – review & editing. WX: Methodology, Writing – review & editing. XL: Writing – review & editing. YD: Writing – review & editing. XX: Writing – review & editing. WY: Conceptualization, Data curation, Writing – review & editing. MC: Writing – review & editing.
