# Supplementary material for: Famine exposure in early life increases risk of cataracts in elderly stage
Source: Front Nutr. 2024 Jun 20;11:1395205. doi: 10.3389/fnut.2024.1395205 (PMC11222645; doi:10.3389/fnut.2024.1395205)
Supplement: Supplementary file 1 [file Image_1.pdf]

Supplementary Material

Supplementary Figures

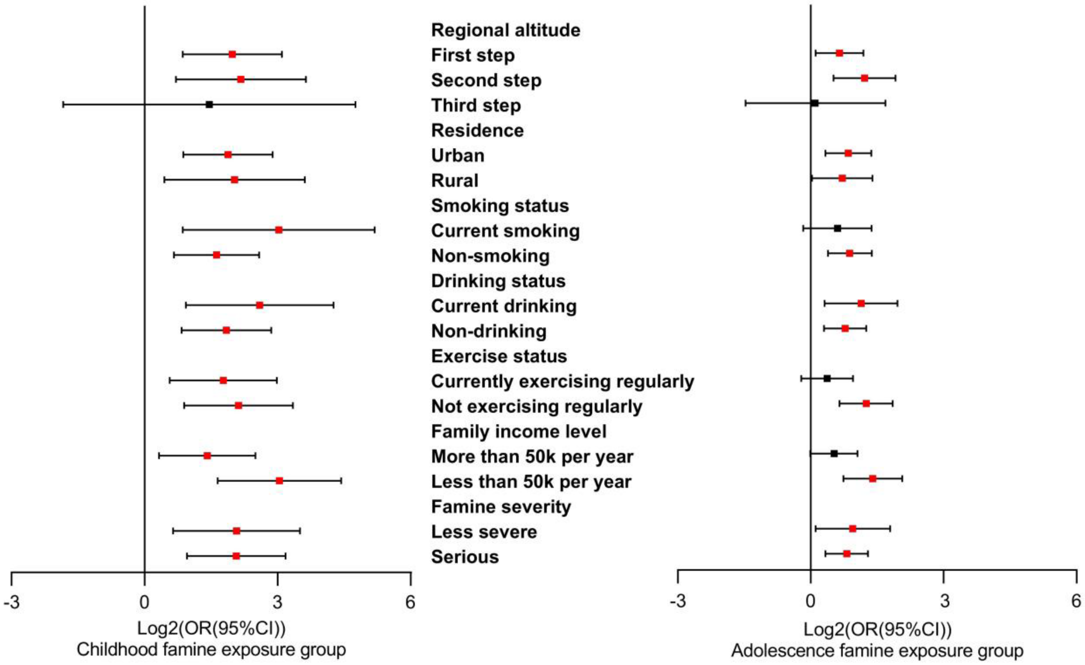

Supplementary Figure 1. Stratified Analysis on Associations between Famine Exposure and Cataracts in Males after Gender Grouping.

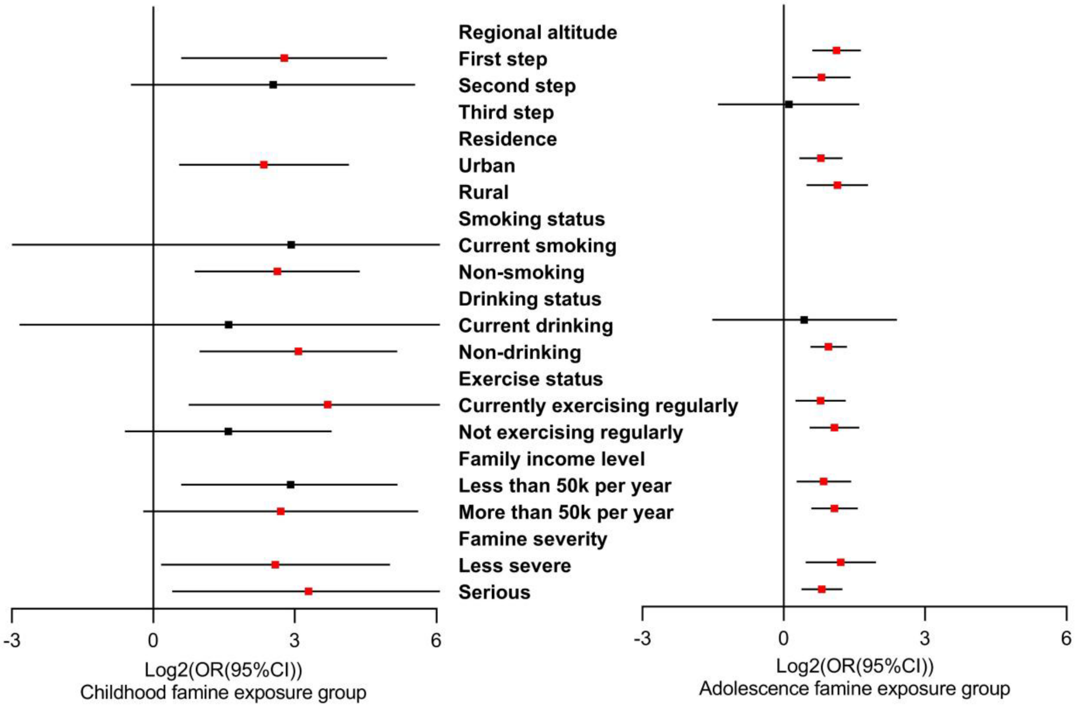

Supplementary Figure2. Stratified Analysis on Associations between Famine Exposure and Cataracts in Females after Gender Grouping.
